# Quantitative Spatial Analysis of Metabolic Heterogeneity Across *in vivo* and *in vitro* Tumor Models

**DOI:** 10.3389/fonc.2019.01144

**Published:** 2019-11-01

**Authors:** Tiffany M. Heaster, Bennett A. Landman, Melissa C. Skala

**Affiliations:** ^1^Department of Biomedical Engineering, University of Wisconsin—Madison, Madison, WI, United States; ^2^Morgridge Institute for Research, Madison, WI, United States; ^3^Department of Electrical Engineering, Computer Engineering, and Computer Science, Vanderbilt University, Nashville, TN, United States

**Keywords:** cell metabolism, tumor heterogeneity, multi-photon microscopy, spatial statistics, image analysis, tumor models

## Abstract

Metabolic preferences of tumor cells vary within a single tumor, contributing to tumor heterogeneity, drug resistance, and patient relapse. However, the relationship between tumor treatment response and metabolically distinct tumor cell populations is not well-understood. Here, a quantitative approach was developed to characterize spatial patterns of metabolic heterogeneity in tumor cell populations within *in vivo* xenografts and 3D *in vitro* cultures (i.e., organoids) of head and neck cancer. Label-free images of cell metabolism were acquired using two-photon fluorescence lifetime microscopy of the metabolic co-enzymes NAD(P)H and FAD. Previous studies have shown that NAD(P)H mean fluorescence lifetimes can identify metabolically distinct cells with varying drug response. Thus, density-based clustering of the NAD(P)H mean fluorescence lifetime was used to identify metabolic sub-populations of cells, then assessed in control, cetuximab-, cisplatin-, and combination-treated xenografts 13 days post-treatment and organoids 24 h post-treatment. Proximity analysis of these metabolically distinct cells was designed to quantify differences in spatial patterns between treatment groups and between xenografts and organoids. Multivariate spatial autocorrelation and principal components analyses of all autofluorescence intensity and lifetime variables were developed to further improve separation between cell sub-populations. Spatial principal components analysis and Z-score calculations of autofluorescence and spatial distribution variables also visualized differences between models. This analysis captures spatial distributions of tumor cell sub-populations influenced by treatment conditions and model-specific environments. Overall, this novel spatial analysis could provide new insights into tumor growth, treatment resistance, and more effective drug treatments across a range of microscopic imaging modalities (e.g., immunofluorescence, imaging mass spectrometry).

## Introduction

Cancer cells within a single tumor have heterogeneous function and phenotype (1), resulting in unpredictable progression and treatment response (2). However, the relationship between these diverse cell populations and global tumor activity is not well-understood. Furthermore, treatment response is altered by interactions between tumor cell populations and their microenvironment. Changes in the tumor microenvironment can contribute to increased tumor cell heterogeneity, directing these cells to adapt genetic, epigenetic, and metabolic processes for growth and survival (3). These cell-level adaptations could include mechanisms of drug resistance, so an understanding of cell-level tumor heterogeneity could provide insight into more effective cancer treatments.

Experimental tumor models are crucial for investigating effects of tumor heterogeneity in cancer progression and drug development. Mouse models are commonly used because they are well-characterized and maintain *in vivo* tumor conditions. 3D organotypic cultures (i.e., organoids) are a popular emerging model system because organoids offer increased throughput compared to *in vivo* models, while maintaining key features of the original tumor, including drug response (4). Both models enable microscopic imaging of tumor cell function and metabolic activity. These models also provide well-defined systems to test new methods for quantifying heterogeneity in tumor cell function. Quantifying spatial functional heterogeneity within *in vivo* mouse models and *in vitro* tumor organoids could establish a link between global tumor drug response and tumor cell heterogeneity, while highlighting differences between *in vivo* and *in vitro* 3D model systems. This link between cell-level behavior and overall tumor response would provide fundamental insight toward developing new treatments that target multiple cell sub-populations, and comparisons between 3D cell culture and *in vivo* systems could inform on the best use of each model system.

Cell-level spatial relationships influence macroscale tumor behavior, but quantitative analysis of tumor microscopic spatial structure has been limited (5). Mathematical modeling has shown promise in simulating tumor spatial heterogeneity but may not account for all biological adaptions that occur within the tumor (6). Alternatively, spatial analysis of experimental models can account for the physical location of observations to quantify local distributions and spatial associations within data, including microscopic images (7). Computational biological image analysis provides quantitative insight into cellular activity (8, 9), and pre-existing data sets provide a readily available source of annotated data to develop and validate these image analysis tools (10–12). A subset of these methods include population clustering, which can identify distinct cell populations within images, and proximity measurements, which define cellular organization within and between these distinct cell populations (13). Spatial autocorrelation also provides a measure of similarity within local cell neighborhoods through comparisons between single cell measurements and averages across neighboring cells, and can be adapted for multivariate assessment (13, 14). Previous studies have used subsets of these techniques to assess qualitative spatial structure within histology sections or fluorescently-labeled samples to describe the organization of multiple cellular compartments and correlate to genetic profiling and prognosis (15–20). However, these approaches can only provide a snapshot of the spatial organization at a single point in time, and require sample destruction, fixation, and labeling. Furthermore, previous studies have not investigated spatial patterns of metabolic heterogeneity at the single cell level within living samples, which may reflect unique sources of microenvironmental stress or drug resistance. Novel processes governing bulk tumor behavior could be characterized by integrating analytical approaches to assess intra-tumor spatial metabolic heterogeneity based on single-cell analysis of viable tumor models.

Tools to assess functional heterogeneity at the cellular level are needed to better understand mechanisms that drive tumor drug response. Optical metabolic imaging (OMI) can non-invasively monitor spatial and temporal changes in cellular metabolism across intact, living 3D tumor models. OMI uses two-photon microscopy to quantify the fluorescence intensities and lifetimes of NAD(P)H and FAD, which are metabolic co-enzymes involved in several cellular metabolic processes (21–23). The fluorescence properties of NADH and NADPH overlap, and are referred to collectively as NAD(P)H. The optical redox ratio, defined as the ratio of NAD(P)H intensity to FAD intensity, measures the oxidation-reduction state of the cell and correlates with mass spectrometry measurements of NADH to NAD+ ratios, and inversely correlates to oxygen consumption measurements (23–28). The fluorescence lifetimes of free and enzyme-bound NAD(P)H and FAD are distinct, and thus provide complimentary information to the optical redox ratio, specifically on enzyme binding activity and quenchers in the microenvironment (23, 25, 26, 29, 30). Previous studies have shown that NAD(P)H lifetimes change depending on the particular enzyme bound to NADH, indicating that that NAD(P)H lifetime reports on shifts in enzyme activity in cells (31). Also, lifetimes of NAD(P)H correlate with intracellular NADPH to NADH concentration ratios (32). OMI has been previously demonstrated for monitoring heterogeneous changes in cell metabolism with drug treatment in mouse models of cancer *in vivo* and in 3D tumor organoids *in vitro* (30, 33, 34). Altogether, OMI generates 3D images at sub-cellular resolution without requiring exogenous labels, sample fixation, or sample sectioning, and thus allows for the 3D spatial context of tumors to be maintained in living samples. Single-cell OMI measurements can quantify metabolic heterogeneity over time and space within the same living sample and can thus relate microscopic heterogeneity to whole-tumor growth.

Here, we developed a suite of spatial statistical analysis tools to quantify the spatial diversity of tumor cell metabolism based on OMI measurements. These tools were applied to previously published *in vitro* and *in vivo* OMI data. Based on previous evidence showing NAD(P)H mean lifetime (τ_m_) identifies distinct tumor cell populations, density-based clustering of NAD(P)H τ_m_ was used here to identify cell populations with distinct metabolic activity within xenografts 13 days post-treatment and organoids 24 h post-treatment (30, 35, 36). Maps of the clustered NAD(P)H lifetime populations were created to qualitatively evaluate the organization of sub-populations and visualize connectivity within and between populations. Population proximity calculations provided quantitative metrics to describe the spatial distribution of NAD(P)H lifetime sub-populations within xenografts and organoids. Multivariate spatial autocorrelation was then designed for all OMI variables to improve separation between metabolic sub-populations based on distinct spatial organization. Finally, z-score calculations and multivariate spatial principal components analysis across all OMI variables were used to assess sample variability and inter-model comparisons of spatial metabolic trends. This work provides a novel approach to quantify spatial patterns in cell function across *in vivo* and *in vitro* tumor models with broad applicability to additional single-cell imaging datasets, such as microscopy images using fluorescent probes or imaging mass spectrometry (37–39).

## Materials and Methods

Recurring abbreviations are listed in Supplementary Table 1.

### Head and Neck Cancer Xenograft Model

Mouse xenografts were grown and treated as previously described (30). Briefly, FaDu human squamous cell carcinoma cells were injected subcutaneously in both flanks of nude mice to generate FaDu xenografts for imaging experiments. Tumor-bearing mice received intraperitoneal injection of vehicle, cetuximab (33 mg/kg) (40, 41), cisplatin (6 mg/kg) (42), or their combination three times per week over 13 days. Tumor growth curves show that cetuximab or cisplatin treatment alone results in stable disease (no change in tumor volume) and their combination results in response (reduction in tumor volume), compared to control over this 13 day treatment time-course (36). Mice from each treatment group were selected for imaging 13 days post-treatment. Flank tumors in anesthetized mice were exposed by cutting away the skin layer covering the tumor prior to transfer onto the microscope stage for *in vivo* imaging. All animal studies were approved by the Vanderbilt University Animal Care and Use Committee and were designed according to NIH animal welfare guidelines. Mice were isoflurane-anesthetized prior to any reported surgical or imaging procedures.

### Organoid Generation

FaDu organoids were generated according to previously reported methods (36). FaDu tumors from nude mice were excised and dissociated to generate cell suspensions for 3D cultures. Macrosuspensions were combined with Matrigel at a 1:2 ratio by volume and plated in 100 μL droplets on 35 mm glass bottomed imaging dishes (MatTek). Organoids were incubated overnight to solidify, then maintained in fresh media prior to start of treatment. At 24 h prior to imaging, media was replaced with treatment-supplemented media containing 20 nM cetuximab (43), 33 μM cisplatin (44, 45), or their combination. Twenty four hours of these treatments results in no significant change in number of cells per organoid or organoid volume. Previous studies demonstrate early treatment response in organoids (1–3 days) is consistent with measured tumor growth at later timepoints (several weeks) (33, 34). Accordingly, 24 h of treatment in organoids does result in significant decreases in NAD(P)H τ_m_ for cisplatin, cetuximab, and cisplatin+cetuximab (combination) treatment, which is an early indicator of treatment response consistent with later changes in tumor volume (36).

### OMI Image Acquisition

Measurements of fluorescence lifetime (FLIM) and intensity were acquired through a two-photon microscope and collected with a GaAsP photomultiplier tube equipped for time-correlated single photon counting (Becker and Hickl). NAD(P)H (750 nm) and FAD (890 nm) fluorescence were excited with a tunable titanium-sapphire laser (Coherent). Fluorescence emission for NAD(P)H and FAD were collected at 400–480 and 500–600 nm, respectively. Intensity and FLIM images were acquired for each field of view with 256 × 256 pixel resolution. Lifetime decay curves were integrated over a 60 s total scan time with a pixel dwell time of 4.8 microseconds. The photon count rate was maintained at ~2–3 × 10^∧^5 photons/second during imaging for optimal photon counting and minimal photobleaching. Each xenograft or organoid was imaged as previously described (30, 46). For xenograft experiments, 4–6 representative fields of view were acquired per tumor ~20–40 μm from the tumor surface, with 2–6 tumors per treatment group (~1,000–2,000 cells per treatment group). For organoid experiments, a single image was captured several cell layers from the surface for 4–6 individual organoids within a given treatment group (~200–400 cells per treatment group). These imaging planes were chosen for both *in vivo* and *in vitro* imaging to avoid surface artifacts and sample a viable region away from the necrotic core.

### Image Analysis

Fluorescence lifetimes corresponding to free and protein-bound NAD(P)H and FAD were calculated using SPCImage (Becker and Hickl). Measured fluorescence decay curves were deconvolved from the instrument response and fit to the following bi-exponential model (26): $I\left(t\right)=\;\alpha_{1}e^{-t/\tau_{1}}+$ $\alpha_{2}e^{-t/\tau_{2}}+C$. Second harmonic generation signal from urea crystals at an incident wavelength of 900 nm was measured to determine the instrument response function (full width at half maximum = 244 ps). From this model, the short and long lifetime components (τ_1_, τ_2_) and the fractional contributions of each (α_1_, α_2_) were calculated for individual pixels across NAD(P)H and FAD images. Photon events over a 3 × 3 pixel area were binned to improve photon count. NAD(P)H and FAD intensity images were generated through pixel-by-pixel integration of photon count over fluorescence decay time for respective lifetime images. The per-pixel ratio of NAD(P)H fluorescence intensity to FAD intensity was then calculated to determine the optical redox ratio. A customized CellProfiler pipeline was used to segment individual cell cytoplasms (nucleus excluded) (35). This mask was applied to all cells per image to compute the redox ratio, mean fluorescence lifetime of NAD(P)H (τ_m_ = α_1_τ_1_ + α_2_τ_2_), FAD τ_m_, free lifetime (τ_1_ for NAD(P)H and τ_2_ for FAD), protein-bound lifetime (τ_2_ for NAD(P)H and τ_1_ for FAD) and fractional contributions of the lifetimes (α_1_ and α_2_) for each cell cytoplasm per image (33). Note that α_1_ + α_2_ = 1 so the fraction can be determined from α_1_ only. Therefore, “OMI variables” include 9 total variables: redox ratio, NAD(P)H τ_m_, τ_1_, τ_2_, α_1_, and FAD τ_m_, τ_1_, τ_2_, α_1_.

### Quantitative Spatial Analysis

**Figure 1:**
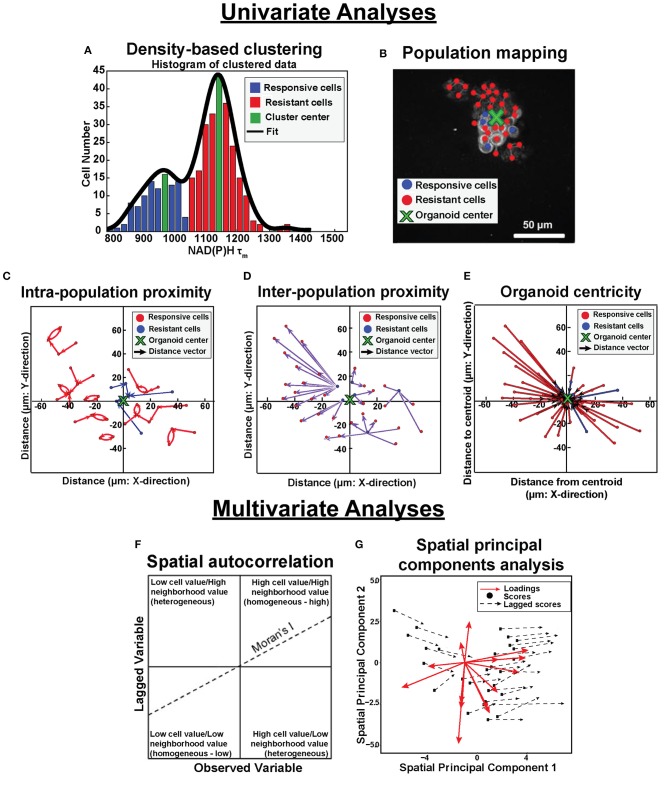
Population density analysis and spatial statistical analysis quantify spatial heterogeneity of cell metabolism. Single variable analyses using NADH τ_m_ data is outlined in **(A–E)**. **(A)** Frequency histograms of single-cell NAD(P)H τ_m_ values (blue, green, red bars) are fit with kernel density fitting (black line) to represent the data distribution. Density-based clustering analysis of NAD(P)H τ_m_ identifies sub-populations based on cluster centers (green bins). Cells are assigned to the nearest cluster with higher local density (responsive cluster, blue bins; resistant cluster, red bins). **(B)** Population spatial maps of NAD(P)H τ_m_-defined clusters include markers of responsive cells (blue dots), resistant cells (red dots), and organoid centroid (green x) on top of the original NAD(P)H intensity image (gray). **(C)** Intra-population proximity is defined as the distance between cells within a population (responsive or resistant), represented by the average length of the blue (responsive) or red (resistant) arrows in the plot. **(D)** Inter-population proximity is defined as the distances between cells belonging to separate populations (responsive or resistant), represented by the average length of the purple arrows in the plot for the responsive (blue) cells. **(E)** Organoid centricity is defined as the distance from the organoid center to each cell within a class (responsive or resistant), represented by the average length of the blue (responsive) or red (resistant) arrows in the plot. Multivariate analyses performed across all OMI variables is described in **(F,G)**. **(F)** Multivariate spatial autocorrelation assesses the similarity of a local cluster by plotting each OMI variable for each cell (observed variable) against the average of its neighboring cells (lagged variable). The slope of the data represents the Moran's I, a global measure of spatial autocorrelation. **(G)** Multivariate spatial principal components analysis illustrates variation between xenografts and organoids as a function of treatment group and cell population (responsive or resistant). Loadings vectors represent the contribution of each OMI or spatial variable to each spatial principal component (x- and y- axes). Scores for each image are calculated from a linear combination of each variable weighted by their loadings. Lagged scores correspond to the combination of weighted variables for neighbors within an image. Vector lengths represent the average magnitude of difference between cell scores and neighbor (lagged) scores.

Analytical tools were developed to quantify spatial distributions of metabolic sub-populations based on OMI variables within *in vitro* and *in vivo* tumor models. These tools quantified the effects of treatment on the spatial diversity of tumor cell metabolism. Through this approach, clustering techniques identified functionally distinct cell populations in OMI data. Additionally, spatial statistical analysis of OMI data revealed patterns in their spatial organization. Analysis steps are briefly outlined in Figure 1 and discussed in detail below.

#### Single Variable Analysis of NAD(P)H τ_m_ Images

##### Cell sub-population assignment and mapping

Previous studies have shown that NAD(P)H τ_m_ can predict drug sensitivity in FaDu tumors and organoids, where low NAD(P)H τ_m_ indicates response to treatment and high NAD(P)H τ_m_ indicates resistance (30, 36). Therefore, the single variable analysis here focuses on NAD(P)H τ_m_. In the current study, NAD(P)H τ_m_ measurements were aggregated across all cells per condition prior to clustering. Kernel density estimation of the data distribution was first used to visualize the presence of multiple cell populations based on NAD(P)H τ_m_ (Figure 1A, black line; “ksdensity” in MATLAB) (47). Density-based clustering methods were then used to detect multiple cell populations within images (“densityClust” in MATLAB) (48). Cell clusters were determined from single-cell NAD(P)H τ_m_ measurements (36). Similarity matrices were calculated from pairwise differences between NAD(P)H τ_m_ values (picoseconds) across all cells within an aggregated dataset. A local density per cell and similarity threshold between cells was calculated as described in Rodriguez and Laio (48). Cluster centers were defined as cells with a high local density and large pairwise difference from the nearest high density cell. Each cell was then assigned to a cluster matching the cluster assignment of the nearest high density cell. Color-coded frequency distribution histograms visualized the bins containing cluster centers (Figure 1A, green bars) and separation of populations (Figure 1A, red and blue bars). Clusters with lowest NAD(P)H τ_m_ were designated as a “responsive” cell population while clusters with highest NAD(P)H τ_m_ were designated as a “resistant” cell population based on previous studies relating drug response to NAD(P)H τ_m_ values (30, 36). Density-based clustering analysis was validated by comparing class assignments of two lines of breast carcinoma cells to expert cell classification based on morphology [expert classification described in (35)]. Confusion matrices and classification accuracy (≥93%, Supplementary Figure 1) confirm the accuracy of density-based clustering.

##### Spatial proximity analysis

Proximity measures were quantified to assess the spatial distribution of cell populations within individual images. Two cells with an inter-cellular physical distance less than the average cell diameter (d_cell_diameter_) were defined as connected neighbors and given a weight of 1, while two cells with inter-cellular distances greater than d_cell_diameter_ (non-neighboring cells) were weighted as 0. Weights were calculated pairwise across all cells within an image. To incorporate population assignments from density-based clustering, weights were kept as 1 if the cell neighbor was defined as responsive or weighted as 2 if the neighbor was defined as resistant. These weights were also defined pairwise for each cell-neighbor combination. Distance matrices were generated to assess the physical distance between neighbors with identical and dissimilar population assignments (i.e., responsive or resistant). The minimum intra-population and inter-population physical distances (i.e., intra- and inter-population proximity) were then determined for each cell, representing the distance to the nearest cell of identical and dissimilar population assignment (Figures 1C,D). Intra-population and inter-population distances were averaged across all cells for a given treatment for both model systems. To account for difference in scale between xenografts and organoids, xenograft distance measurements were normalized to the width of the imaging field of view, while organoid measurements were normalized to the organoid diameter. Additionally, centroids were calculated for each organoid sample (“regionprops” in MATLAB) to evaluate physical distance from each cell to the organoid center. Distance to organoid centers (i.e., organoid centricity) was independently assessed for responsive and resistant cell populations (Figure 1E).

#### Multivariate Spatial Analysis of All OMI Variable Images

Multivariate analyses were used to determine cellular spatial organization across all OMI variables (redox ratio, NAD(P)H τ_m_, τ_1_, τ_2_, α_1_, and FAD τ_m_, τ_1_, τ_2_, α_1_) for improved separation between responsive and resistant cells.

##### Multivariate spatial autocorrelation

Spatial autocorrelation of all OMI variables was evaluated at the global and local scale for all samples (xenografts and organoids). Moran's I (Figure 1F) was used to determine macroscale spatial similarity for each OMI variable per image (ape package in R) (49). Moran's I was defined as:

(1) $$\begin{array}{l} I=\;\frac{N}{W}\frac{\sum_{i}^{i=m}\sum_{j}^{j=n}w_{ij}\left(x_{i}-\bar{x}\right)\left(x_{j}-\bar{x}\right)}{\sum_{i}^{i=m}{\left(x_{i}-\bar{x}\right)}^{2}} \end{array}$$

Where *w*_*ij*_ represents the weight (0 or 1) indicating connection (1) or no connection (0) between a pair of cells, *x*_*i*_, *x*_*j*_ represent the OMI variable value at a given cell location, $\bar{x}$ represents the OMI variable average across the image, *N* represents the number of cells in the image, and *W* represents the total sum of the weight matrix (w_m,n_) (14).

The range of Moran's I values extended between −1 and 1. An image containing cells surrounded by cells with similar OMI variable values is represented by I values approaching 1, while an image with cells surrounded by cells with dissimilar OMI variable values is represented by I values approaching −1. Images with cells that are surrounded by cells with both similar and dissimilar OMI variable values (i.e., random organization) is represented by I values near 0. All spatial autocorrelation analysis was implemented in R (ape, ade4 packages in R) (49, 50).

Local indicators of spatial association (LISA) were used to visualize the similarity of OMI variables within local cell neighborhoods as a function of model system (xenograft or organoid), treatment condition, and drug response (responsive or resistant) (51). For each OMI variable, individual cell measurements (Figure 1F, x-axis) were correlated with the measurement average of its “neighbors” (cells within one cell diameter) (Figure 1F, y-axis). A cell was defined as “high” or “low” if the OMI variable value for the cell was above or below the mean across all cells within an image, respectively (Figure 1F, vertical line). Similarly, a neighborhood (defined as all the neighbors of a given cell) was defined as “high” or “low” if the mean OMI variable for the neighborhood was above or below the mean across all neighborhoods within an image, respectively (Figure 1F, horizontal line) (51). Individual cells and their neighbors with identical definitions (high/low) for a given OMI variable were designated as homogeneous cell neighborhoods (Figure 1F, upper right and lower left quadrants). High prevalence of homogeneous cell neighborhoods indicated metabolic activity was largely dependent on spatial organization of cells.

##### Spatial principal components analysis

Spatial principal components analysis (spatial PCA) was applied by modifying standard principal components analysis to account for spatial structure based on Moran's I statistics (adespatial package, R) (34, 35, 52). All OMI variables and spatial parameters collectively were referred to as “variables.” Lag matrices were created from the product of spatial weights matrices (w_m,n_, Equation 1) and corresponding variable matrices. Covariance between the variable matrix and lagged variable matrix was then assessed to define the principal component axes. The spatial principal component loadings were determined, representing each variable's contribution to a linear combination maximizing both the variance and Moran's I across the data. Loading vectors were plotted to observe the magnitude and direction of each variable projected onto the spatial principal component axes (Figure 1G, red arrows) (53). Principal component scores per image were determined by averaging linear combinations of the product of each variable and the corresponding component loadings across all cells (Figure 1G, black dots). Similarly, lagged scores were determined by averaged linear combinations of the lag matrix weighted by the spatial principal component loadings (Figure 1G, arrowheads). Vectors connecting principal component scores and lagged scores demonstrated the average difference in measurements between cells and their neighbors for a given condition.

##### Z-score standardization

The magnitude of differences between xenografts and organoids was assessed by compiling variables (OMI variables and spatial parameters) across all experimental groups to compare Z-scores across models. Z-score transformation for all variables per image was performed by subtracting the variable average and dividing by the variable standard deviation of the corresponding control or treated organoid condition (54). Z-score heatmaps were generated across all treatment conditions and model systems to demonstrate differences between models (gplots package, R) (55).

### Statistical Analysis

Student's *t*-tests and Tukey's multiple comparison statistical tests for non-parametric, unpaired comparisons were performed to assess differences across organoid and xenograft treatment conditions (54). Error bars indicate the mean ± standard deviation. Measurements with an alpha value <0.05 were considered statistically significant. Cohen's d values were also computed to determine effect size (54). Local constant non-parametric regression was used to assess significant relationships between treatment condition and Moran's I for each OMI variable (56, 57). Xenograft or organoid treatment condition and corresponding standard error of Moran's I values served as explanatory variables evaluated for effects on the dependent variable, Moran's I.

## Results

### Spatial Clustering Based on NAD(P)H τ_m_

To distinguish spatial differences in heterogeneous cell populations with respect to treatment, we first implemented spatial clustering based on a single OMI variable, specifically NAD(P)H τ_m_. This was assessed on a previously published dataset of images from control and cetuximab-, cisplatin-, and combination-treated FaDu xenografts and organoids after 13 days and 24 h, respectively. Published studies of this dataset include standard measurements of organoid and *in vivo* treatment response, which are consistent with studies showing agreement between early treatment response in organoids and long-term tumor volume measurements (33, 34, 36). NAD(P)H τ_m_ was chosen for this analysis as decreases in NAD(P)H τ_m_ with treatment *in vivo* and in organoids correlate with later decreases in FaDu tumor volume (30). Cells with high NAD(P)H τ_m_ were treatment resistant, whereas cells with low NAD(P)H τ_m_ were responsive to treatment in these previous studies. Later analysis (**Figures 4–6**) defined multivariate spatial heterogeneity across all 9 OMI variables.

**Figure 2:**
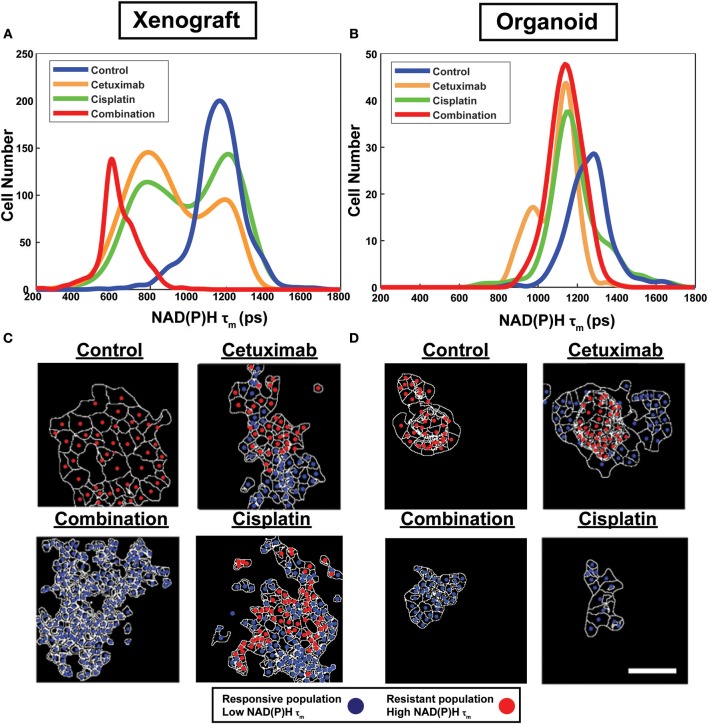
Sub-population distributions and population mapping visualize treatment-dependent spatial heterogeneity within FaDu xenografts and organoids. Population density modeling of single cell measurements of NAD(P)H τ_m_ in FaDu **(A)** xenografts and **(B)** organoids reveal heterogeneous cell populations within control, cetuximab-treated, cisplatin-treated, or cetuximab and cisplatin-treated (combination) groups. Representative population maps of control and treated **(C)** xenografts and **(D)** organoids demonstrate spatial organization of cell populations with differing treatment response. Individual cells are color-coded based on the population assignment determined from density-based clustering analysis. Responsive populations, corresponding to low NAD(P)H τ_m_, are coded red, and resistant populations, corresponding to high NAD(P)H τ_m_, are coded blue. Cell outlines are in white. Scale bar, 50 μm.

Population distributions were used to visualize the presence of multiple cell populations with either high (resistant) or low (responsive) NAD(P)H τ_m_ in response to treatment (Figure 2). Sub-population analysis also demonstrated the extent of heterogeneity across models and treatment groups (Figures 2A,B). For example, multiple populations are only present in cetuximab-treated organoids but are present in both cetuximab- and cisplatin-treated xenografts, suggesting xenografts may exhibit increased heterogeneity in response to single agent treatment (Figures 2A,B).

Density-based clustering was performed to classify treatment response on a single-cell level. Density-based classification using NAD(P)H τ_m_ was validated with high accuracy (≥93%; Supplementary Figure 1) compared to expert classification in 2D cultures of cell lines. Following validation, this method was used to classify responsive and resistant cells within xenografts and organoids. Population assignments for each cell were mapped back to the images to display the spatial organization of responsive and resistant populations (Figures 2C,D). Population maps qualitatively demonstrate low dispersion of cell populations and their macroscale organization. Overall, visualization of cell organization revealed spatial clustering patterns of resistant and responsive cell populations, which are unique to specific treatment and microenvironmental conditions.

**Figure 3:**
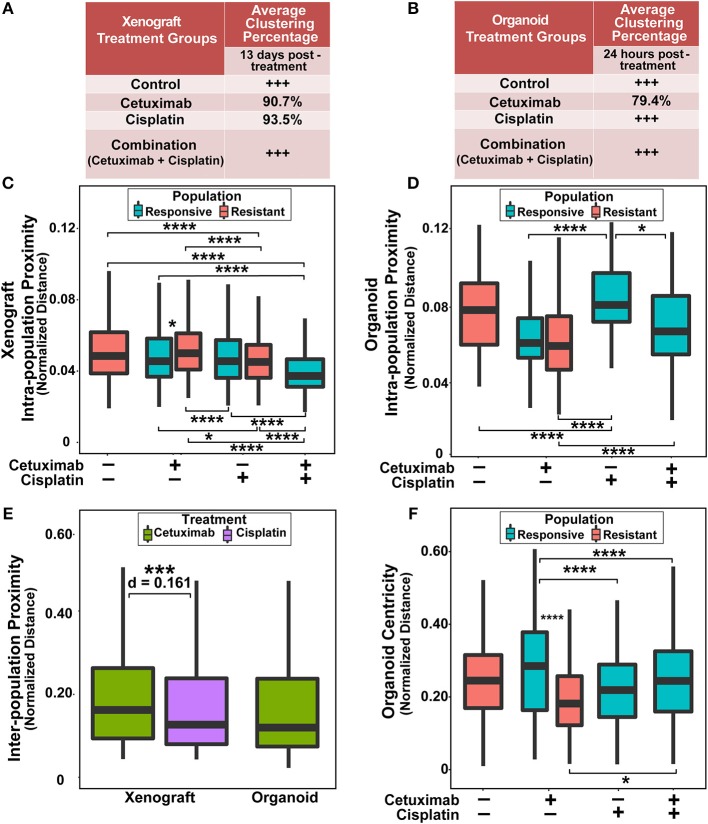
Spatial clustering and proximity based on NAD(P)H τ_m_ for responsive and resistant cell populations. **(A)** Clustering percentage in xenografts after 13 days of treatment. Control and combination-treated xenografts have only one population of cells each, indicated by +++. **(B)** Clustering percentage in organoids after 24 h of treatment. Only cetuximab treatment yields multiple cell populations in organoids. **(C)** Average distance between cells within a single population (intra-population proximity) in xenografts. **(D)** Intra-population proximity in organoids. **(E)** Average distance between responsive and resistant cells (inter-population proximity) in xenografts and organoids for treatments that have 2 populations. **(F)** Average distance to organoid centers (organoid centricity). (*, ***, ****p < 0.05, 0.001, 0.0001; Tukey-HSD test).

Next, quantitative metrics were developed to directly compare the spatial distributions of responsive and resistant cell populations that were defined by NAD(P)H τ_m_ and evaluated on control and drug-treated FaDu xenograft and organoid images (Figure 3). Clustering percentages informed on mixing between responsive and resistant cells, providing an objective comparison of cell dispersion across treatment conditions and model systems (Figures 3A,B). For example, xenografts 13 days post-treatment displayed considerable segregation between responsive and resistant cells, with >90% of cell neighbors belonging to the same population in conditions with both cell classes (cetuximab and cisplatin groups; Figure 3A), while 24 h treated organoids yielded lower clustering (~80%) of responsive and resistant cell populations (cetuximab group only, Figure 3B).

Intra-population proximity measurements demonstrated density of cell packing within each population (Figures 3C,D). Highly compact cell organization was represented by low intra-population distances, demonstrated by the single, responsive cell population in combination-treated xenografts (Figure 3C, *p* < 0.0001). Conversely, high intra-population distances illustrated sparse cell organization, reflected through increased distances in cisplatin-treated organoid populations compared to control, cetuximab, and combination populations (Figure 3D, *p* < 0.0001). Responsive populations in cetuximab-treated xenografts formed denser clusters than resistant populations (*p* < 0.05), while no significant differences were observed between responsive and resistant cells with cisplatin treatment (Figure 3C). However, cisplatin-resistant cell populations had more compact cell organization than resistant cells in control xenografts (*p* < 0.001). Additionally, cisplatin treatment resulted in closer proximity within resistant xenograft populations than cetuximab treatment (Figure 3C, *p* < 0.001). Intra-population proximity measurements also provided comparisons of both density and uniformity of population clusters, most distinct between cetuximab and cisplatin organoids (Figure 3D, *p* < 0.05). Spatial organization of cell sub-populations appeared to be independent of sample size, as significant differences in organoid diameter measurements were only observed between control and cetuximab-treated organoids despite unique spatial organization across all groups (Supplementary Figure 2).

Inter-population distances quantified localization of cell populations relative to each other, comparable across treatment groups with multiple cell populations (Figure 3E). Inter-population distances were not significantly different between xenograft and organoid cells treated with cetuximab, suggesting that cell populations organize similarly in cetuximab-treated organoids and xenografts (Figure 3E). However, cisplatin-treated xenografts had decreased inter-population distances compared to cetuximab-treated xenografts (Figure 3E, *p* < 0.001).

Additionally, organoid centricity measurements provided assessment of cell organization relative to central and peripheral organoid regions (Figure 3F). This served as a complementary measure of cell packing in organoids with only one cell population (control, cisplatin, and combination groups), indicating similar packing across these conditions (Figure 3F, *p* > 0.05). Furthermore, organoid centricity measurements showed resistant populations within cetuximab-treated organoids aggregated closer to organoid centers than responsive cells (Figure 3F, *p* < 0.0001). Responsive populations in cetuximab-treated organoids localized further from the organoid center compared to responsive populations in cisplatin and combination organoids, demonstrating a change in distribution of responsive cells when multiple sub-populations are present (Figure 3F, *p* < 0.0001). Heatmaps comparing spatial parameters between resistant and responsive populations across treatment conditions demonstrate direction of change, significance, and effect size (Supplementary Figure 3).

### Spatial Clustering Based on Multivariate Analysis of All 9 OMI Variables

Distinct clustering was demonstrated for responsive and resistant cell populations defined by a single variable, NAD(P)H τ_m_ (Figure 3). However, multivariate analysis was used to determine spatial patterns of the 8 other OMI variables (redox ratio, NAD(P)H τ_1_, τ_2_, α_1_, and FAD τ_m_, τ_1_, τ_2_, α_1_) in attempt to improve separation between responsive and resistant tumor cell populations. Multivariate measures of spatial autocorrelation for all OMI variables were quantified to comprehensively assess metabolic relationships between single cells and their surrounding neighbors. Macroscale clustering for each OMI variable was determined by calculating global Moran's I per OMI variable for control and treated FaDu xenograft and organoid images.

**Figure 4:**
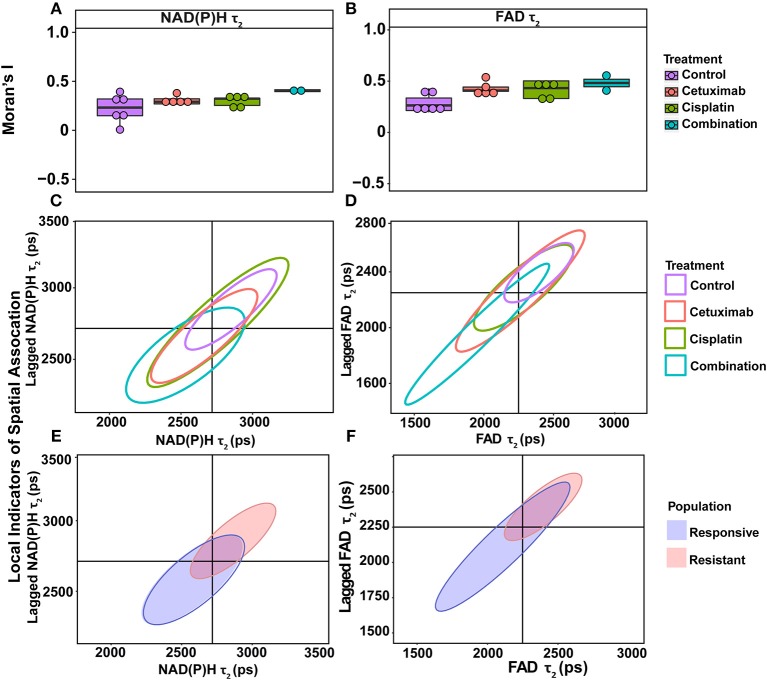
Spatial clustering patterns of OMI variables in FaDu xenografts. Moran's I based on **(A)** NAD(P)H τ_2_ and **(B)** FAD τ_2_ measurements is plotted across control and treated xenografts. **(C–F)** Ellipses represent the distribution of cell clusters, with cells falling in the upper right (homogeneously high value clusters) and lower left quadrants (homogeneously low value clusters). Clustering is plotted for each treatment group for **(C)** NAD(P)H τ_2_ and **(D)** FAD τ_2_. Clustering is similarly plotted for responsive and resistant cells for **(E)** NAD(P)H τ_2_ and **(F)** FAD τ_2_.

**Figure 5:**
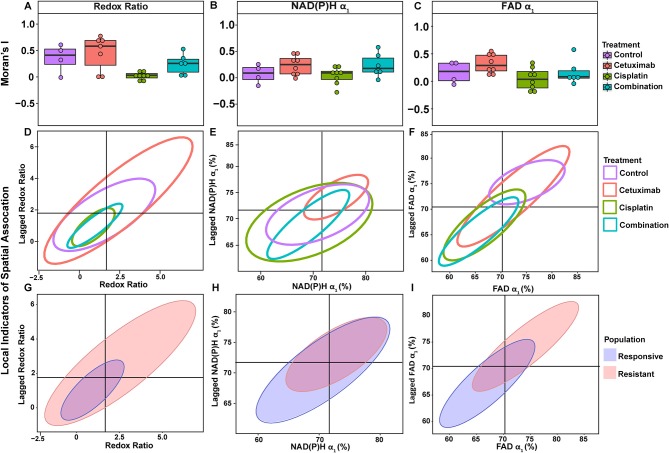
Spatial clustering patterns of metabolic parameters in FaDu organoids. Moran's I based on **(A)** redox ratio, **(B)** NAD(P)H α_1_, and **(C)** FAD α_1_ measurements for control and treated organoids. **(D–F)** Ellipses represent the distribution of cell clusters, with cells falling in the upper right (homogeneously high value clusters) and lower left quadrants (homogeneously low value clusters). Clustering is plotted for each treatment group for **(D)** redox ratio, **(E)** NAD(P)H α_1_, and **(F)** FAD α_1_. Cluster patterns of responsive and resistant cells are plotted for **(G)** redox ratio, **(H)** NAD(P)H α_1_, and **(I)** FAD α_1_.

Moran's I was first calculated for each OMI variable across all treatment conditions, then non-parametric regression was performed to determine which OMI variables yielded Moran's I values with significant dependence on treatment condition (Tables 1, 2). Regression analysis identified NAD(P)H and FAD τ_2_ as significant variables for FaDu xenografts and redox ratio, NAD(P)H and FAD α_1_ for FaDu organoids. Similarity in clustering patterns within treatment groups was represented by low variability in Moran's I, observed for all OMI variables across all xenografts per treatment group (Figures 4A,B, Supplementary Figure 4). Conversely, organoids demonstrated greater variability of Moran's I within treatment groups (Figures 5A–C, Supplementary Figure 7). Positive spatial autocorrelation indicated increased cluster formation, displayed in virtually all xenograft treatment groups across all OMI variables (Figures 4A,B, Supplementary Figure 4). Overall, combination treatment demonstrated the highest Moran's I values for τ_2_ measurements, while control xenografts were characterized by low Moran's I (Figures 4A,B). Random organization of metabolic activity was illustrated by Moran's I values near zero, reflected in redox ratio, NAD(P)H α_1_ and FAD α_1_ of cisplatin-treated organoids (Figures 5A–C). Conversely, cetuximab treatment resulted in the greatest Moran's I of these variables, indicating clusters of homogenous metabolic activity within cetuximab-treated organoids (Figures 5A–C). Furthermore, cisplatin treatment consistently yielded Moran's I near zero across all OMI variables, while other organoid treatment conditions displayed varied response (Supplementary Figure 7).

**Table 1:** *P*-values from non-parametric regression between treatment condition and Moran's I in xenografts.

**OMI Variable**	***P*-value**	**OMI Variable**	***P*-value**
Redox Ratio	0.11	FAD τ_m_	0.383
NAD(P)H τ_m_	0.323	FAD τ_1_	0.707
NAD(P)H τ_1_	0.183	**FAD** **τ_2_**	**0.015**
**NAD(P)H** **τ_2_**	**0.043**	FAD α_1_	0.378
NAD(P)H α_1_	0.246	FAD Intensity	0.155
NAD(P)H Intensity	0.178		

*Bold values represents p < 0.05*.

**Table 2:** *P*-values from non-parametric regression between treatment condition and Moran's I in organoids.

**OMI Variable**	***P*-value**	**OMI Variable**	***P*-value**
**Redox Ratio**	**0.005**	FAD τ_m_	0.331
NAD(P)H τ_m_	0.398	FAD τ_1_	0.248
NAD(P)H τ_1_	0.301	FAD τ_2_	0.494
NAD(P)H τ_2_	0.627	**FAD** **α_1_**	**0.005**
**NAD(P)H** **α_1_**	**0.008**	FAD Intensity	0.175
NAD(P)H Intensity	0.266		

*Bold values represents p < 0.05*.

LISA provided complementary information to Moran's I by describing the relationship between single cells and their local neighborhood (description in Figure 1F). OMI variables per cell (observed variable) were directly compared with the average of surrounding cells (lagged variable). Cell clusters within xenografts predominantly had τ_2_ values that were homogeneously high (control group) or homogeneously low (combination group), represented by cells within the upper right and lower left quadrants, respectively (Figures 4C,D). Additionally, cisplatin- and combination-treated organoids were both predominantly composed of low redox ratio and FAD α_1_ clusters (Figures 5D,F). Higher frequency of cells in the upper left and lower right LISA quadrants demonstrated greater heterogeneity in OMI variables within cell clusters, observed for τ_2_ measurements in cisplatin- and cetuximab-treated xenografts and redox and FAD α_1_ measurements in cetuximab-treated organoids (Figures 4C,D, 5D,F). NAD(P)H α_1_ clusters similarly exhibit substantial heterogeneity across control and treated organoids (Figure 5E). OMI variables not significantly dependent on treatment yielded variable clustering in xenografts and organoids, with some treatment groups primarily restricted to one quadrant (Supplementary Figures 5, 8).

LISA were also used to assess distinct clustering of OMI variables based on treatment response. In xenografts, resistant populations form clusters of high NAD(P)H and FAD τ_2_ (Figures 4E,F). In contrast, responsive populations predominantly organize into clusters with low τ_2_ (Figures 4E,F). Responsive populations are also characterized by clusters of lower NAD(P)H and FAD τ_m_ and τ_2_, and higher NAD(P)H and FAD α_1_ compared with resistant cells (Supplementary Figure 6). Responsive and resistant cells in organoids also had distinct clustering patterns (Figures 5G–I). Responsive cells in organoids were characterized by low redox ratio clusters (Figure 5G). Resistant cells conversely had clusters of higher NAD(P)H and FAD α_1_ compared to responsive cells (Figures 5H,I). Responsive and resistant populations demonstrated variable clustering across other OMI variables (Supplementary Figure 9).

### Metabolic and Spatial Variability in Xenograft and Organoid Models

**Figure 6:**
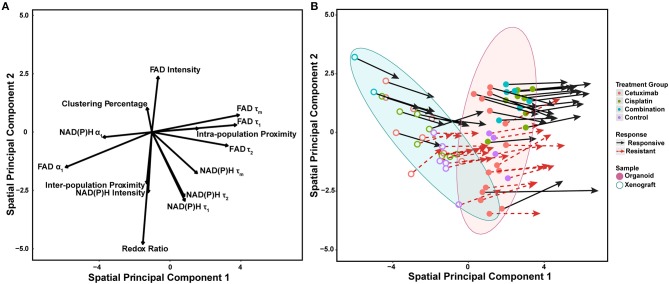
Multivariate analyses of metabolic activity and spatial organization in xenografts and organoids. **(A)** Loading vectors for all treatment conditions across both organoids and xenografts. **(B)** The same spatial PCA plot as **(A)**, with treatment groups and model systems plotted to observe spatial clustering patterns. Points correspond to the spatial PCA score for a single organoid (filled circle) or xenograft (open circle). Arrows represent differences between average spatial PCA scores of an organoid or xenograft and the average spatial PCA score of their cell neighborhood.

Spatial principal components analysis (spatial PCA) was used to relate multivariate measurements of cell metabolism with spatial trends across models and treatment conditions (Figure 6A) (58). Briefly, multivariate metabolic and spatial data was weighted by a binary matrix indicating neighbors for each cell (i.e., cell neighborhood) prior to standard calculation of principal components. FAD τ_1_, τ_2_, τ_m_, and intra-population distances had the highest positive loadings along spatial principal component 1, which reflects their dominant contribution to overall variability within organoids and xenografts (Figure 6A). Both NAD(P)H and FAD α_1_ had the largest negative loadings along spatial principal component 1, which also reflects their dominant, yet opposing contribution to overall variability within organoids and xenografts. NAD(P)H τ_1_, τ_2_, τ_m_, all intensity-based measurements, and two spatial metrics (clustering percentage, inter-population proximity) had stronger projection along the second principal axis. Variable vectors with similar directionality and magnitude are correlated. As expected, NAD(P)H τ_1_, τ_2_, τ_m_, and separately, FAD τ_1_, τ_2_, τ_m_ exhibited strong positive correlation (τ_m_ = α_1_τ_1_ + α_2_τ_2_). The redox ratio [defined as NAD(P)H/FAD intensity] was positively correlated with NAD(P)H intensity, but negatively correlated with FAD intensity. Inter-population proximity was closely related to redox ratio and NAD(P)H intensity, while intra-population proximity was closely related to FAD τ_1_ but not highly correlated with the other two spatial metrics (clustering percentage, inter-population proximity). In contrast, clustering percentage was inversely correlated with inter-population proximity.

Mean spatial PCA scores were then plotted for both responsive and resistant cells within each control and treatment group for both xenografts and organoids to observe similarities across models (Figure 6B). Each point represents the mean spatial PCA score for an organoid (filled) or xenograft (hollow), and points were plotted on the same axes as Figure 6A. The location of each dot can be correlated with metabolic variables on the same axes in Figure 6A. Xenografts were generally correlated with variables in the upper left quadrant of Figure 6A [e.g., NAD(P)H α_1_, clustering percentage] compared to organoids, which correlate with NAD(P)H and FAD τ_1_, τ_2_, and τ_m_ (blue vs. red ellipse, Figure 6B).

Vectors extending from points on spatial PCA plots described the response of cell neighborhoods within the organoid or xenograft (arrows, Figure 6B). The location of the arrowhead represents differences between the average cell response and the average cell neighborhood response. Of note, fewer xenografts were imaged per treatment group, and thus clustering and correlation of cell neighborhoods were less defined compared to the more high-throughput organoid analysis. Mean spatial PCA scores of both cells and their neighborhoods in responsive organoid populations are predominantly correlated within the combination treatment group. Conversely, resistant cells within cetuximab and control organoids are not correlated. Spatial PCA maps at the cellular level across treatments and models (organoids and xenografts) also help to visualize the organization of cell neighborhoods (Supplementary Figure 10).

**Figure 7:**
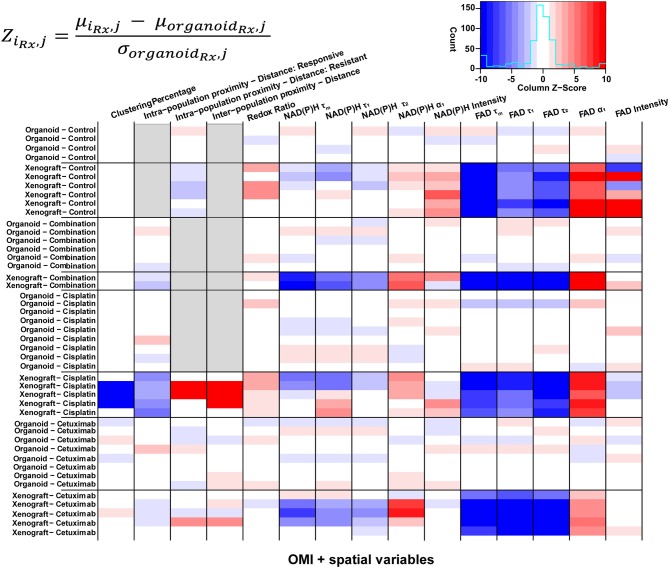
Z-scores of metabolic and spatial variables in control and treated xenografts and organoids. Heatmap of Z-scores across treatment groups and model systems (organoid and xenograft). Each Z-score is calculated for each sample of a given treatment group (i_Rx_) and variable (j) as the difference between the mean variable per sample and the mean variable across all organoids of the corresponding treatment group (organoid_Rx_) divided by the standard deviation of the organoid treatment group. Z-score differences within a single treatment group represent variability across samples. Gray boxes indicate samples without a value for a given variable, specific to xenograft and organoid treatment groups with a single population.

Heatmaps of Z-scores for OMI variables and spatial parameters were generated for further comparison between treatment groups and model systems (Figure 7). Z-scores were calculated relative to the mean and standard deviation of the corresponding organoid condition to visualize differences between models for each treatment group. Xenografts consistently exhibited higher FAD α_1_ and lower FAD τ_m_, τ_1_, and τ_2_ compared with organoids, regardless of treatment condition. Intensity measurements were generally increased in control xenografts compared to control organoids. Additionally, control xenografts exhibited shorter intra-population distances within resistant populations. Organoids and xenografts showed substantial differences in response to combination treatment. Specifically, combination-treated xenografts yielded higher α_1_ and lower τ_m_, τ_1_, and τ_2_ for both NAD(P)H and FAD. This trend was also observed between models in response to cetuximab treatment. Spatial measurements displayed contrast between cisplatin-treated xenografts and organoids due to the presence of two populations in xenografts, compared to a single population in organoids. Cisplatin treatment also caused increased redox ratio and NAD(P)H α_1_ in xenografts compared to organoids. Heatmaps of Z-scores within xenograft and organoid conditions are shown in Supplementary Figure 11.

## Discussion

Spatial heterogeneity within tumors contributes to poor therapeutic response and tumor recurrence. However, tools to characterize intra-tumor heterogeneity at the cellular level and its effect on disease progression have been limited to destructive methods that require tumor dissociation, and thus removal from the host. Here, we have combined OMI with spatial statistical methods to quantify organization of multiple cell sub-populations within intact, heterogeneous samples (35, 47). Previous studies have established that OMI can resolve phenotypic differences within 2D and 3D *in vitro* tumor models, and *in vivo* mouse tumors (23, 24, 27, 30, 33, 36, 59). However, these previous studies did not thoroughly assess spatial distributions of metabolic cell sub-populations, which is important for monitoring cell-cell interactions in tumors (60). We have developed novel analytical methods to quantify cell-level heterogeneity in tumor models by combined OMI and density-based clustering (Figure 1A) (29, 30, 43–45). Density-based clustering methods promote robust identification of distinct, rare populations by exclusively considering distances between data points, circumventing assumptions about the data distribution typically necessary for clustering (48, 61). Spatial statistical analysis of data clustered by a single variable [NAD(P)H τ_m_, previously shown to separate drug-responsive and -resistant cells (12, 26)] provides unique metrics for the organization of heterogeneous cell populations within samples (Figures 1B–E). Finally, multivariate analyses of spatial organization across all OMI variables enables greater separation between responsive and resistant cells to compare spatial trends within a sample (Figures 1F,G). In the future, OMI and quantitative metrics of cellular connectivity could guide the development of novel therapies that target drug-resistant tumor microenvironments for improved therapeutic outcomes.

This is the first study to combine label-free, live cell imaging, and spatial statistical techniques to assess treatment-dependent changes in tumor cell function. Previous studies have used microscopic spatial analyses to determine the organization of phenotypically distinct cell populations within fixed, stained tumor tissue sections (16–20). For example, distributions of tumor and stromal cells at the time of biopsy or surgery have been correlated with clinical outcome, but these studies disregard both heterogeneity within cell types and treatment-specific cell organization (17, 62). Otherwise, previous microscopic work has focused on spatial patterns of genetic expression rather than functional (e.g., metabolic) heterogeneity (63–66). Macroscopic heterogeneity in metabolite concentration and uptake *in vivo* has been previously investigated by PET and MRI, but these techniques lack the spatial resolution to detect cell-level heterogeneity (64, 67). Notably, endogenous metabolic fluorescence imaging has previously evaluated macroscale spatial distributions of the redox ratio *in vivo* and *in vitro* (65). These studies observed higher redox ratio and NAD(P)H intensity tumor regions, but low FAD regions localized at the periphery of untreated mouse breast carcinoma xenografts. Interestingly, we observed the same trend in centrally-located resistant cell populations across organoids (Figures 3F, 5G, Supplementary Figure 9). Unlike these previous studies, our current study quantified cell-level spatial distributions of distinct metabolic sub-populations (Figure 3) and the clustering patterns of these metabolic sub-populations (Figures 4, 5), which varied with respect to treatment and drug response in live, intact, unstained samples.

The spatial distribution of metabolically-distinct cell populations was quantified for samples with distinct treatment and drug response because cell metabolism is both a therapeutic target and a route of tumor evasion (68). Responsive cells were previously shown to exhibit lower NAD(P)H τ_m_ with cetuximab or cisplatin treatment compared to resistant cells, which often emerge from the selective pressures of cancer treatment (30, 36, 69). Clustering percentage (Figures 3A,B), intra-population distances (Figures 3C,D), and inter-population distances (Figure 3E) represented the relative organization of NAD(P)H τ_m_-defined populations in xenografts and organoids. These results showed that cells with similar treatment response (i.e., responsive/resistant) pack together closely, and that cells formed single, uniform clusters of responsive or resistant cells, as opposed to several small, dispersed clusters. Spatial analysis of all OMI variables indicated high intercellular dissimilarity and more random cluster patterns in organoids compared to xenografts (Figures 4, 5, Supplementary Figures 5–8), in contrast to clustering based on NAD(P)H τ_m_ alone (Figures 2D, 3D–F), which indicates that spatial analysis of all OMI variables together captures different metabolic features than NAD(P)H τ_m_ individually. However, spatial heterogeneity was low in control and combination-treated groups compared to single-agent treatment for both xenografts and organoids (Figures 4C,D, 5D–F, Supplementary Figures 5, 8). Though spatial patterns were only quantified for single plane images at defined time points in this study, this analysis can be translated to data acquired over multiple sample depths and treatment time points to determine their influence on cellular spatial organization. Overall, this spatial analysis highlights substantial differences in the local metabolic landscape of combination and single agent treatments across tumor models.

Notably, NAD(P)H lifetime can be modulated in response to alternative metabolic and environmental changes, highlighting the complexity associated with functional readouts of cell metabolism (32, 70, 71). Cellular response to treatment can also modulate several subcellular metabolic processes, collectively reflected by changes in NAD(P)H lifetime (72). Consequently, further development of this analysis to incorporate environmental and functional measurements would be valuable for resolving biological mechanisms driving spatial changes in cellular behavior. For example, proximity to vasculature could be inferred from metabolic autofluorescence images as blood vessels appear as dark, branch-like regions. Biomarkers of local environmental conditions (e.g., oxygen/pH sensors, metabolic enzyme assays) could be correlated with spatial patterns in OMI data. Future work can also integrate our spatial metabolic analysis with microfluidic models of microenvironmental gradients to disentangle relationships between nutrient availability, environmental stressors, and treatment response (73). Collectively, integration of this spatial analysis with complementary biological data could provide additional insight into mechanisms altering NAD(P)H lifetime and associated drug sensitivity, leading to more effective treatment regimens (74).

Previous studies confirm that xenografts and organoids have consistent treatment response across multiple model systems, using standard measures of response (e.g., tumor volume, cell viability) (75–79). Published studies also show that NAD(P)H τ_m_ decreases with cetuximab or cisplatin treatment in both FaDu xenografts and organoids (30, 36). The current paper develops spatial analysis to quantify differences in cellular spatial organization between models and treatment conditions. In these datasets, xenografts had decreased FAD τ_m_, τ_1_, τ_2_ and increased FAD α_1_ compared to organoids, regardless of spatial distribution (Figure 7). This difference in FAD lifetimes averaged across all cells could be due to a number of factors, including different imaging time-points post-treatment, although organoids provide an early measure of response (1–3 days post-treatment) that agrees with later tumor volume (weeks post-treatment), which motivated the imaging time points used in this study (33, 34). Differences in FAD lifetimes could also be due to shifts in metabolic flux, enzyme binding activity, temperature, oxygenation, and/or pH *in vivo* compared to *in vitro* (76–78).

We also demonstrate that spatial analysis can capture differences in the distribution of cellular drug response between *in vivo* and *in vitro* systems. Specifically, responsive populations defined by NAD(P)H τ_m_ in xenografts were more densely clustered than resistant populations, while the opposite trend was observed in organoids (Figures 3C,D). Also, the spherical geometry of organoids maintain responsive cells on the periphery and resistant cells clustered in the core (Figure 3F), consistent with previous reports (80). The observed differences in the distribution of cell metabolism between models are likely due to distinct methods of drug delivery between organoids (diffusion) and xenografts (vascular delivery). Organoids require drug diffusion from the outer to inner cell layers, which act as a physical barrier limiting penetration of both small and large molecule drugs (~100 Da−1 kDa) and cause gradations in drug dose and metabolic activity (81). In contrast, poor vascularity and inefficient tumor microvessels in xenografts result in a complete lack of drug dosing for some cells *in vivo* (82). This poor vascularity could contribute to segregation of cell populations with distinct metabolic activity *in vivo* (Figures 4A–C, Supplementary Figure 5). Spatial patterns of cellular drug response could be correlated with drug diffusion to assess the influence of drug accessibility on cellular metabolic distributions. Overall, these differences in drug delivery between models highlight the utility of each model for studies of drug efficacy and drug delivery. Notably, organoids are useful for high-throughput and time-course measurements of drug efficacy at early time-points using numerous drugs, whereas xenografts can model both drug delivery and treatment effects. Although drug delivery in xenografts may only partially reflect drug delivery *in vivo* human tumors, xenografts still serve as a useful first approach (83, 84).

The methods developed in this study establish the combination of OMI and spatial statistical analysis to quantify the spatial heterogeneity of tumor cell metabolism. We have shown that cell-level spatial organization of metabolism is altered by treatment and model system. These methods could be translated to OMI data acquired over an entire organoid volume or a superficial tumor volume to characterize 3D distributions of metabolism and drug response. In addition, this analytical approach could integrate complementary metrics from autofluorescence images including cell morphology, intracellular metabolic changes, and other endogenous fluorophores. Furthermore, this approach can be extended to or combined with other single cell imaging approaches probing as gene (e.g., RNA or DNA-FISH) and protein expression (e.g., immunofluorescence) (85, 86). Overall, cell-level functional imaging and quantitative analysis of spatial heterogeneity could significantly improve understanding of tumor growth and treatment resistance.

## Data Availability Statement

The raw data supporting the conclusions of this manuscript will be made available by the authors, without undue reservation, to any qualified researcher.

## Ethics Statement

The animal study was reviewed and approved by Vanderbilt University Animal Care and Use Committee.

## Author Contributions

TH designed and performed the analysis, interpreted the data, made the figures, and drafted the manuscript. BL and MS helped design the analysis, interpreted the data, and edited the manuscript.

### Conflict of Interest

The authors declare that the research was conducted in the absence of any commercial or financial relationships that could be construed as a potential conflict of interest.
