# Supplementary material for: Quantitative Spatial Analysis of Metabolic Heterogeneity Across in vivo and in vitro Tumor Models
Source: Front Oncol. 2019 Nov 1;9:1144. doi: 10.3389/fonc.2019.01144 (PMC6839277; doi:10.3389/fonc.2019.01144)
Supplement: Supplementary file 1 [file Data_Sheet_1.pdf]

Supplementary Material

1 Supplementary Figures and Tables

1.1 Supplementary Figures

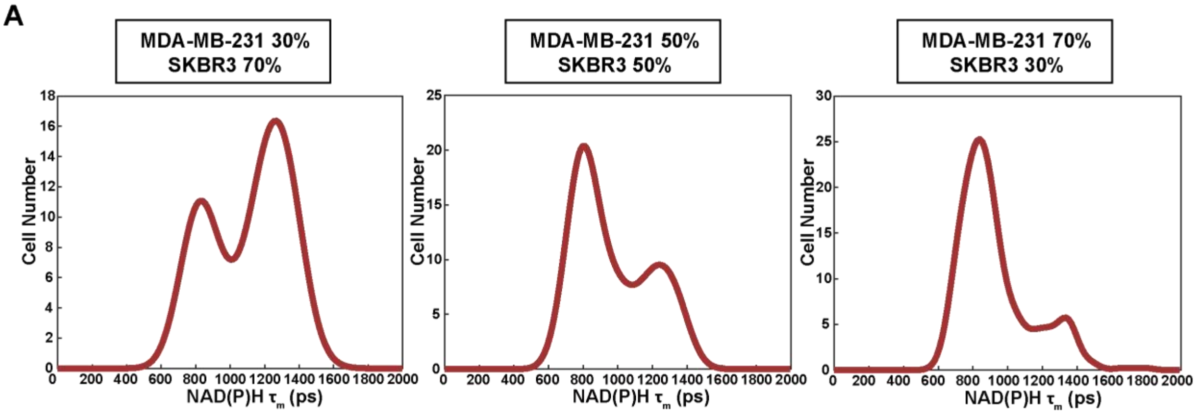

B

Predicted	Actual					
	MDA 30%:SKBR3 70%		MDA 50%:SKBR3 50%		MDA 70%:SKBR3 30%	
	MDA-MB-231	SKBR3	MDA-MB-231	SKBR3	MDA-MB-231	SKBR3
MDA-MB-231	67	3	129	5	220	5
SKBR3	5	92	9	58	7	41
**Accuracy**	**95.2%**		**93.0%**		**95.6%**	

Supplementary Figure 1. Validation of density-based clustering of cell sub-populations. (a) Population density modeling using kernel density estimation visualizes cell sub-populations in breast carcinoma co-cultures plated at known proportions. (b) Classification with density-based clustering analysis demonstrates high accuracy for all co-culture conditions compared to actual classification (manual classification by an expert based on morphology [35]) analysis demonstrates high accuracy for all co-culture conditions compared to actual classification (manual classification by an expert based on morphology [35])

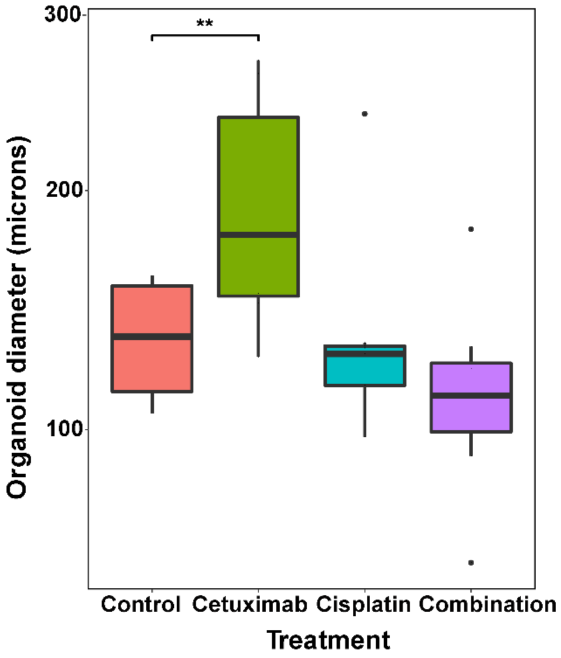

Supplementary Figure 2. Organoid diameter measurements across control and treated conditions. Organoid diameter is calculated for each control organoid and cetuximab-, cisplatin- and combination-treated organoids. Control organoids are smaller in diameter compared with cetuximab-treated organoids. Diameter differences between all other treatment conditions lack significance. (**p <0.01; Tukey-HSD test).

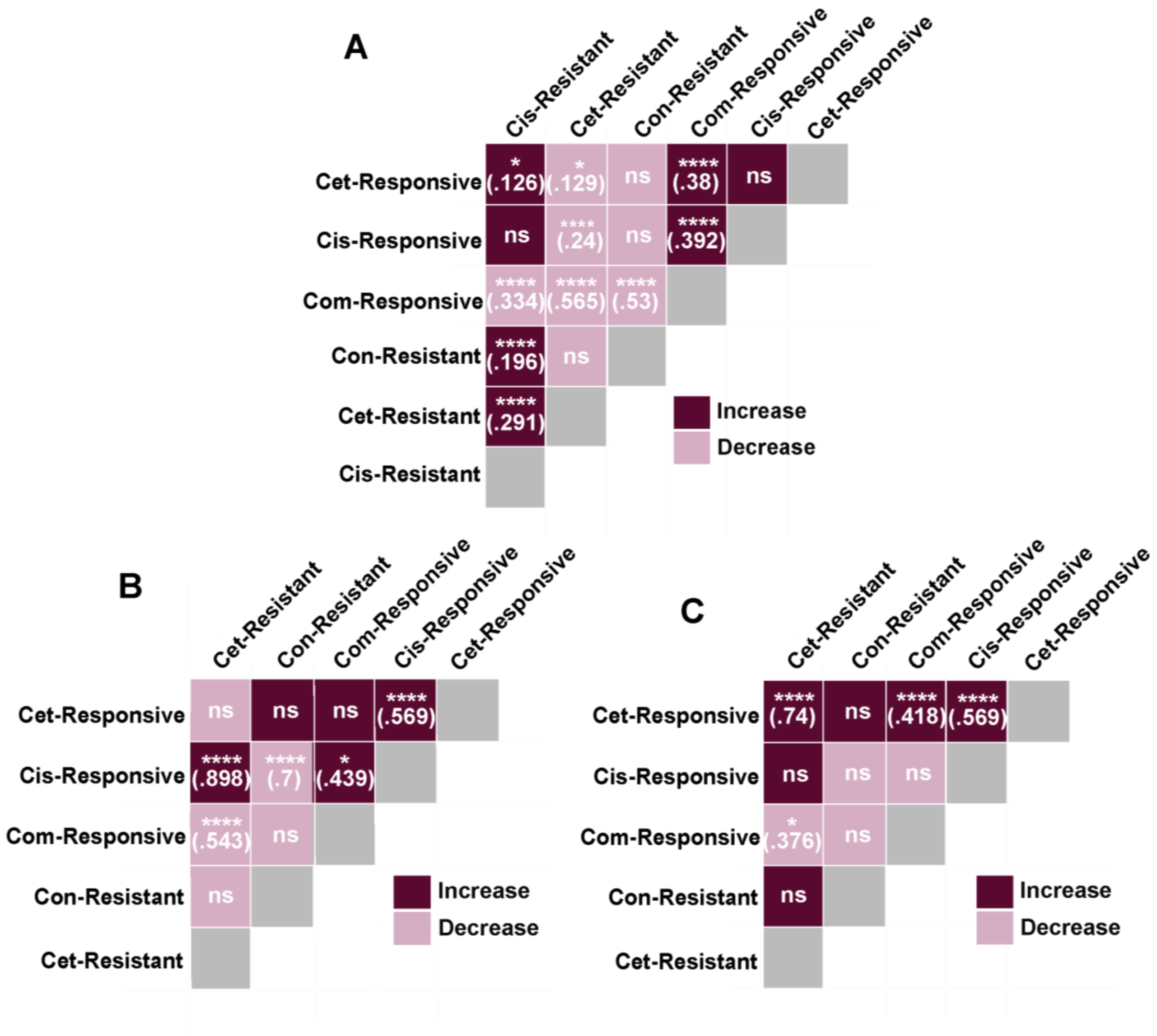

Supplementary Figure 3. Significance and effect size heatmaps reveal differences in spatial parameters between responsive and resistant populations in control and treated xenografts and organoids. Heatmaps show the direction of change, significance, and effect size for row conditions with respect to the column conditions. Statistical significance (asterisks) and effect size (notated values) of (a) xenograft intra-population proximity, (b) organoid intra-population proximity, and (c) organoid centricity is calculated between responsive and resistant populations in each treatment condition. (*, ****p <0.05, 0.0001; Tukey-HSD test). Con = control; Cet = cetuximab; Cis = cisplatin; Com = combination.

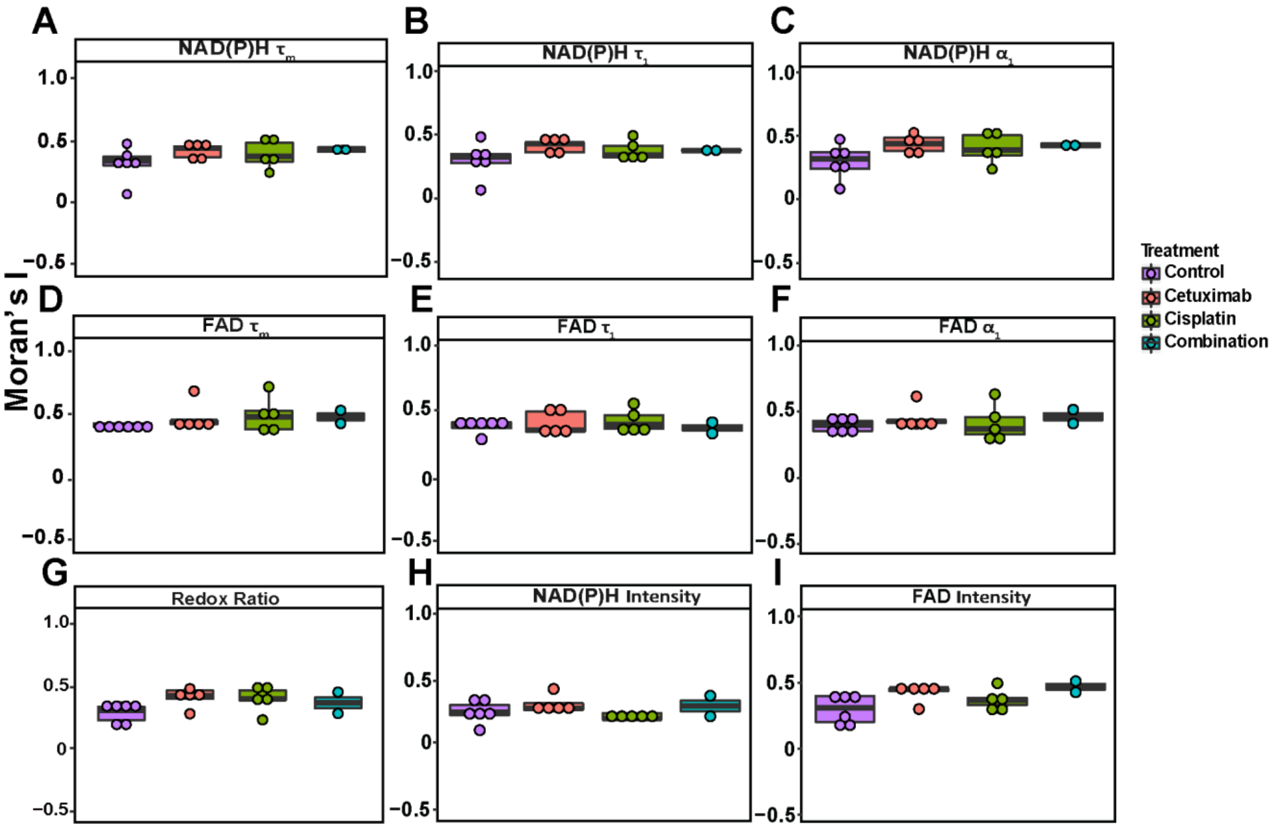

Supplementary Figure 4. Global Moran's I for NAD(P)H and FAD intensity and fluorescence lifetime components in control and drug-treated xenografts. Moran's I is calculated for each xenograft across treatment conditions for the following OMI variables: (a) NAD(P)H τ_m, (b) NAD(P)H τ_1, and (c) NAD(P)H α_1, (d) FAD τ_m, (e) FAD τ_1, (f) FAD α_1, (g) redox ratio, (h) NAD(P)H intensity, and (i) FAD intensity. Positive spatial autocorrelation ($I \gg 0$) is observed for all intensity and lifetime measurements for each treatment condition.

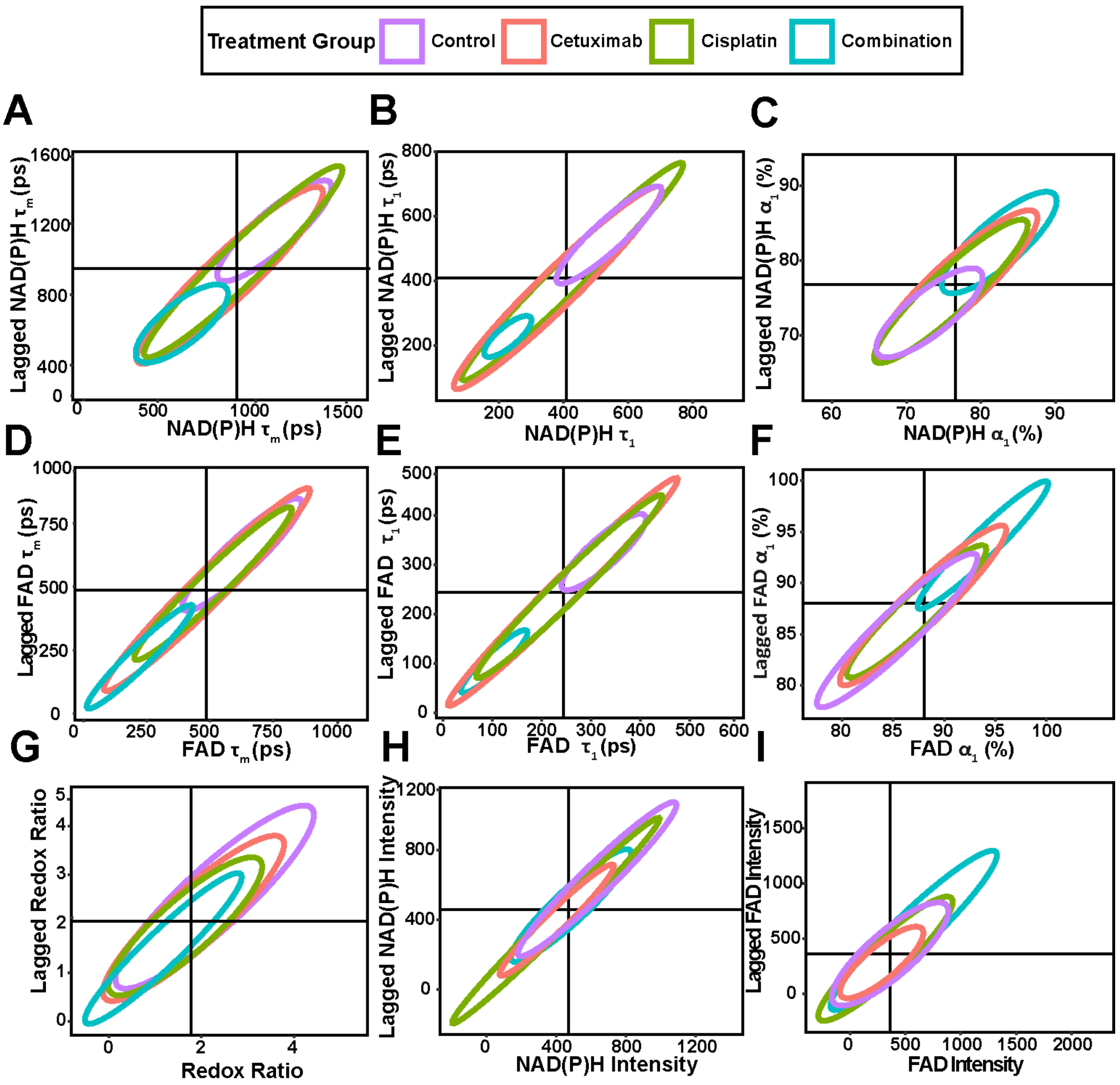

Supplementary Figure 5. Local indicators of spatial association (LISA) for NAD(P)H and FAD intensity and lifetime components across xenograft treatment groups. Unique clustering patterns are observed for (a) NAD(P)H τ_m, (b) NAD(P)H τ_1, (c) NAD(P)H α_1, (d) FAD τ_m, (e) FAD τ_1, (f) FAD α_1, (g) redox ratio, (h) NAD(P)H intensity, and (i) FAD intensity measurements between control and cetuximab-, cisplatin-, and combination-treated xenografts.

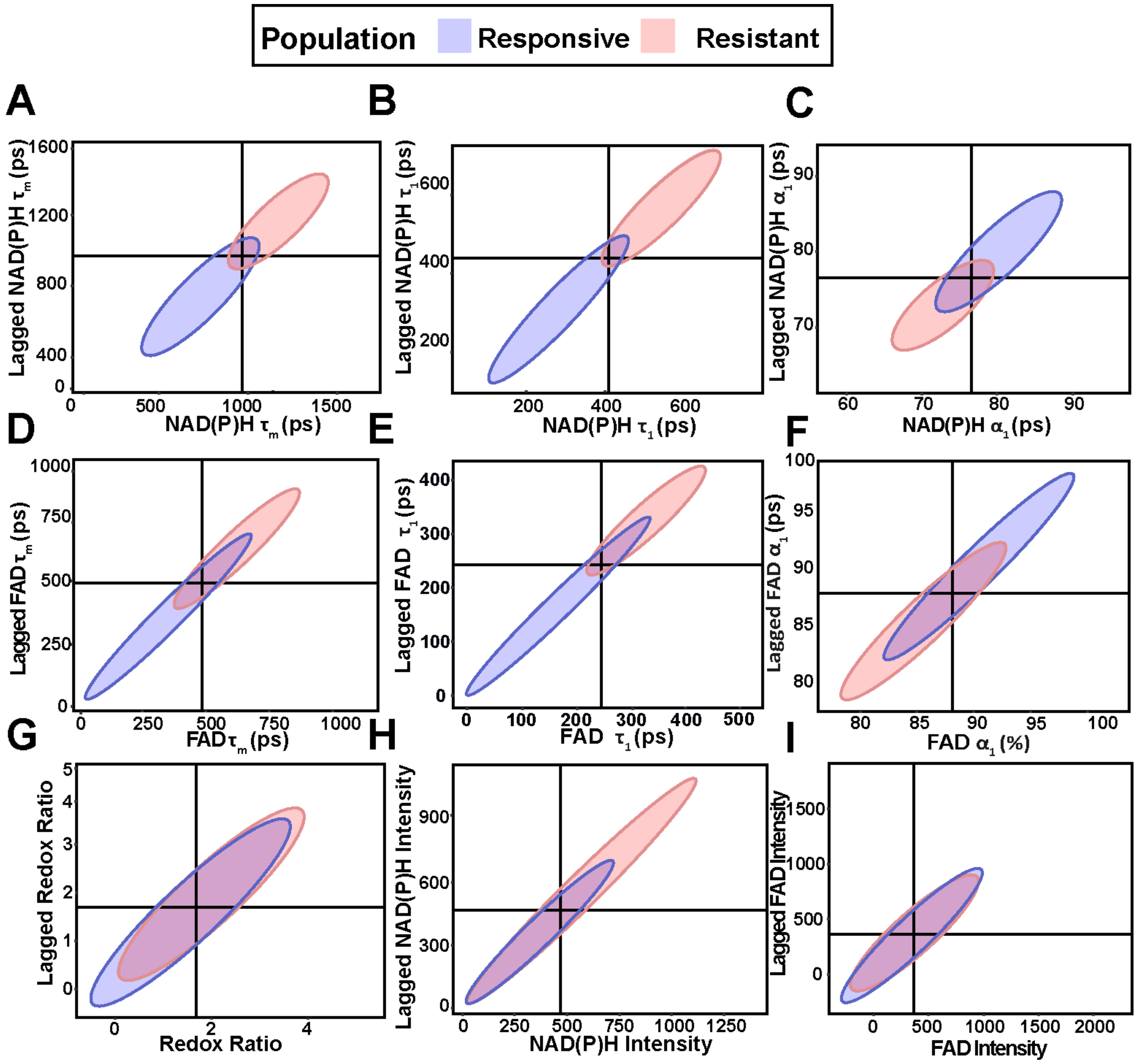

Supplementary Figure 6. Local indicators of spatial association (LISA) for NAD(P)H and FAD intensity and lifetime components for responsive and resistant xenograft populations. Clustering patterns for responsive and resistant populations are shown for (a) NAD(P)H τ_m, (b) NAD(P)H τ_1, (c) NAD(P)H α_1, (d) FAD τ_m, (e) FAD τ_1, (f) FAD α_1, (g) redox ratio, (h) NAD(P)H intensity, and (i) FAD intensity measurements.

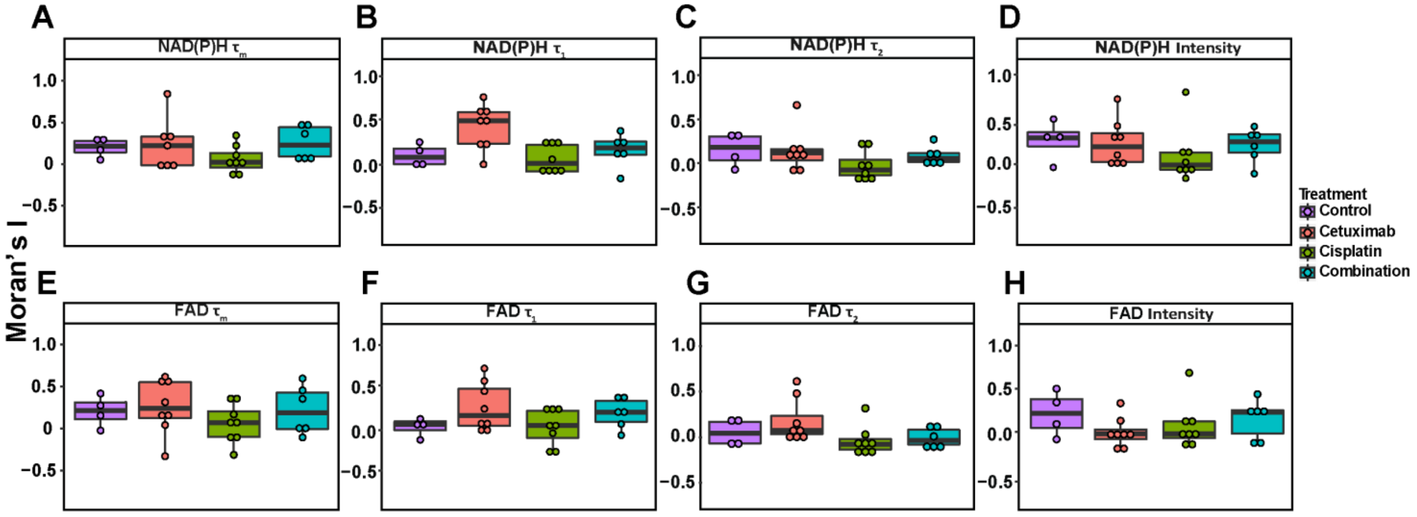

Supplementary Figure 7. Global Moran's I for NAD(P)H and FAD intensity and fluorescence lifetime components in control and drug-treated organoids. Organoids across treatment groups exhibit minimal spatial autocorrelation ($0.5 > I > 0$) for (a) NAD(P)H τ_m, (b) NAD(P)H τ_1, (c) NAD(P)H τ_2, and (d) NAD(P)H intensity measurements, as well as (e) FAD τ_m, (f) FAD τ_1, (g) FAD τ_2, and (h) FAD intensity measurements

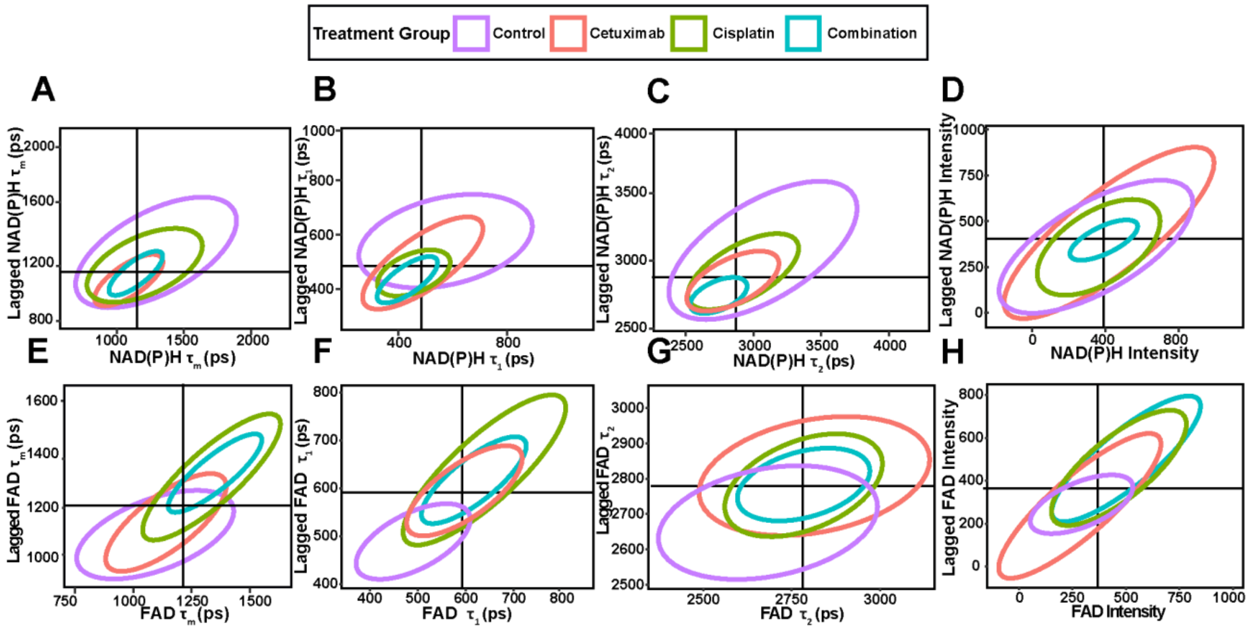

Supplementary Figure 8. Local indicators of spatial association (LISA) for NAD(P)H and FAD intensity and lifetime components across organoid treatment groups. Differences in clustering of (a) NAD(P)H τ_m, (b) NAD(P)H τ_1, (c) NAD(P)H τ_2, (d) NAD(P)H intensity, (e) FAD τ_m, (f) FAD τ_1, (g) FAD τ_2, and (h) FAD intensity measurements are observed between control organoids and organoids treated with cetuximab, cisplatin, or their combination.

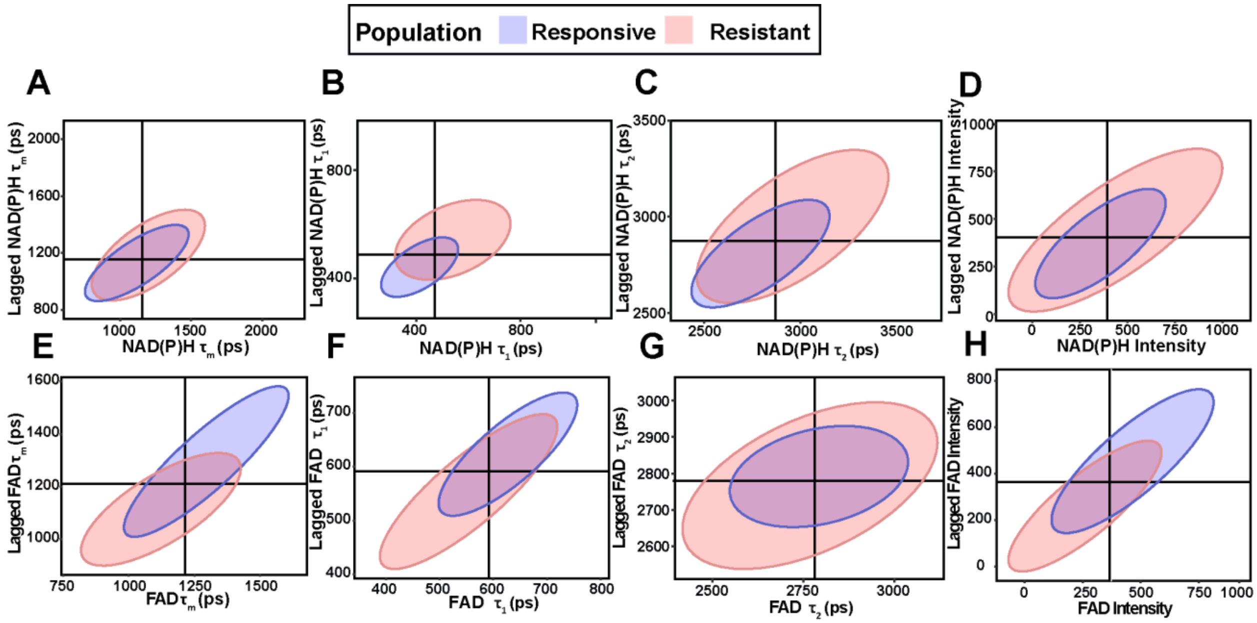

Supplementary Figure 9. Local indicators of spatial association (LISA) for NAD(P)H and FAD intensity and lifetime components for responsive and resistant organoid populations. Responsive and resistant populations exhibit characteristic clustering of (a) NAD(P)H τ_m, (b) NAD(P)H τ_1, (c) NAD(P)H τ_2, and (d) NAD(P)H intensity measurements, as well as (e) FAD τ_m, (f) FAD τ_1, (g) FAD τ_2, and (h) FAD intensity measurements.

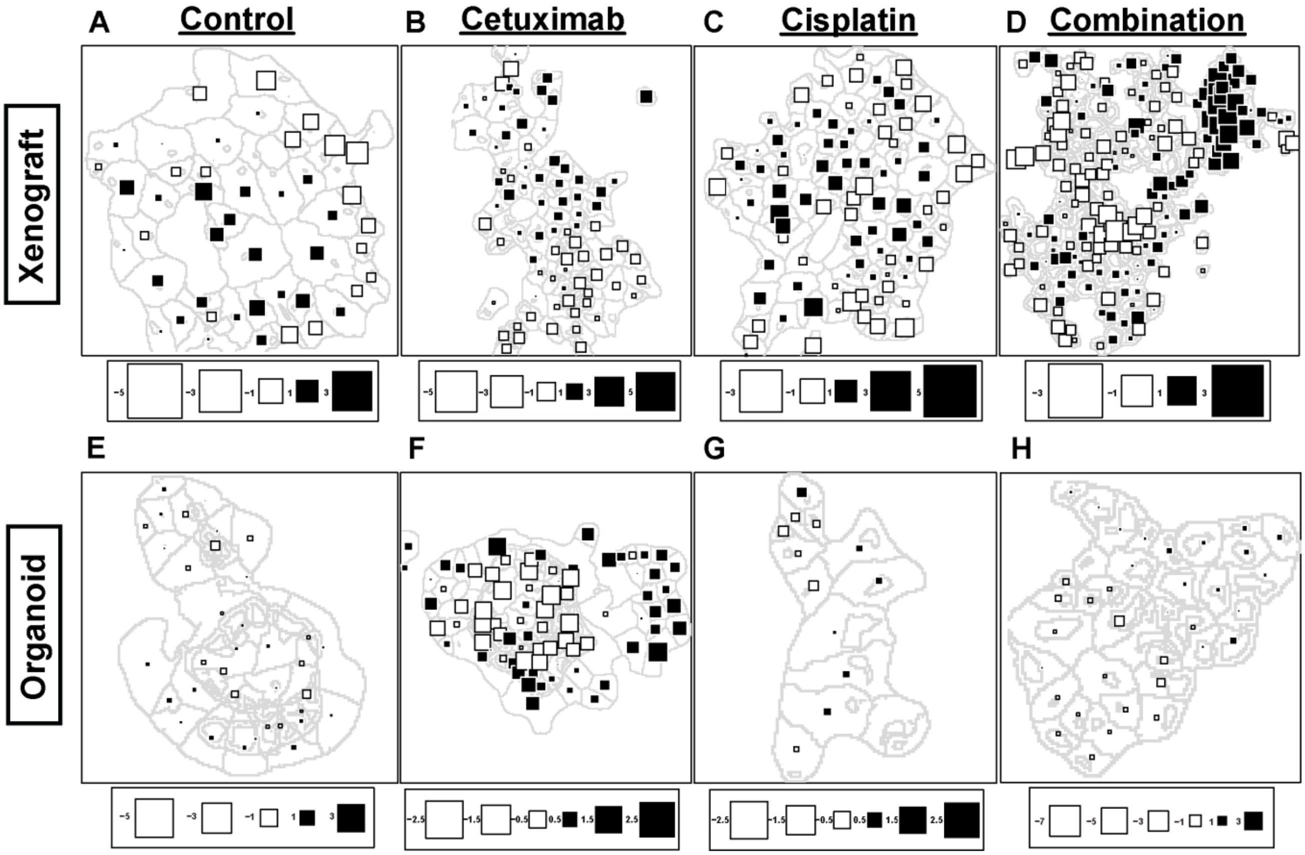

Supplementary Figure 10. Representative spatial principal components analysis (SPCA) maps. Individual SPCA scores along the first principal axis are mapped to corresponding cells within xenografts (a-d) or organoid (e-h) images. Square size represents magnitude of the score. Positive scores are color coded black, while negative scores are color coded white.

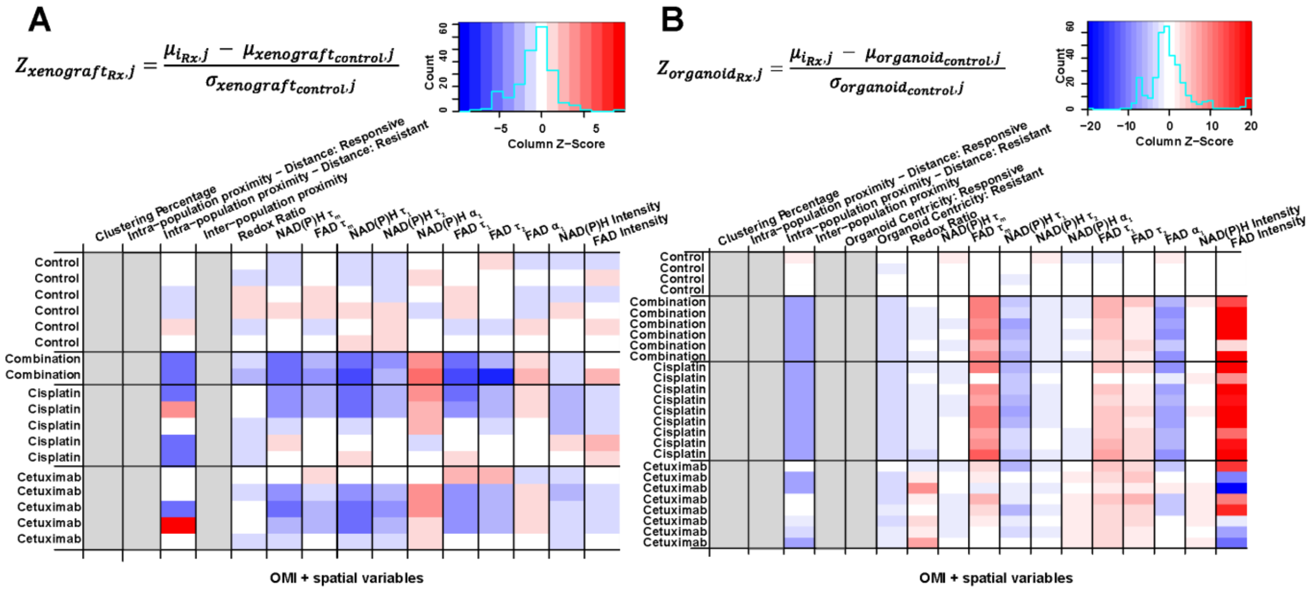

Supplementary Figure 11. Assessment of inter-sample variability across treatment groups within xenograft and organoid models. OMI and spatial variables for each (a) xenograft and (b) organoid sample were Z-score transformed with respect to the corresponding control sample averages and plotted as heatmaps to visualize relative differences between control and treated samples. Gray boxes indicate samples without a value for a given variable, due to lack of variation across the control samples.

1.2 Supplementary Tables

Supplementary Table 1: List of Abbreviations

Abbreviation	Explanation
OMI	Optical metabolic imaging
FLIM	Fluorescence lifetime imaging
τ_m	Mean lifetime
τ_1	Short lifetime
τ_2	Long lifetime
α_1	Fractional component
$d_{cell\_diameter}$	Average cell diameter
spatial PCA	Spatial principal components analysis
LISA	Local indicators of spatial association
